# Using ancient sedimentary DNA to forecast ecosystem trajectories under climate change

**DOI:** 10.1098/rstb.2023.0017

**Published:** 2024-05-27

**Authors:** Inger Greve Alsos, Victor Boussange, Dilli Prasad Rijal, Marieke Beaulieu, Antony Gavin Brown, Ulrike Herzschuh, Jens-Christian Svenning, Loïc Pellissier

**Affiliations:** ^1^ The Arctic University Museum of Norway, UiT The Arctic University of Norway, 9037 Tromsø, Norway; ^2^ Department of Environmental System Science, ETH Zürich, Universitätstrasse 16, 8092 Zürich, Switzerland; ^3^ Swiss Federal Research Institute WSL, Zürcherstrasse 111, 8903 Birmensdorf, Switzerland; ^4^ Alfred Wegener Institute for Polar and Marine Research, Telegraphenberg A43, 14473 Potsdam, Germany; ^5^ Institute of Environmental Sciences and Geography, Potsdam University, 14479 Potsdam, Germany; ^6^ Center for Ecological Dynamics in a Novel Biosphere (ECONOVO) & Center for Biodiversity Dynamics in a Changing World (BIOCHANGE), Department of Biology, Aarhus University, Ny Munkegade 114, 8000 Aarhus C, Denmark

**Keywords:** modelling, sedimentary ancient DNA, forecast, ecosystem, biodiversity, time series

## Abstract

Ecosystem response to climate change is complex. In order to forecast ecosystem dynamics, we need high-quality data on changes in past species abundance that can inform process-based models. Sedimentary ancient DNA (*sed*aDNA) has revolutionised our ability to document past ecosystems' dynamics. It provides time series of increased taxonomic resolution compared to microfossils (pollen, spores), and can often give species-level information, especially for past vascular plant and mammal abundances. Time series are much richer in information than contemporary spatial distribution information, which have been traditionally used to train models for predicting biodiversity and ecosystem responses to climate change. Here, we outline the potential contribution of *sed*aDNA to forecast ecosystem changes. We showcase how species-level time series may allow quantification of the effect of biotic interactions in ecosystem dynamics, and be used to estimate dispersal rates when a dense network of sites is available. By combining palaeo-time series, process-based models, and inverse modelling, we can recover the biotic and abiotic processes underlying ecosystem dynamics, which are traditionally very challenging to characterise. Dynamic models informed by *sed*aDNA can further be used to extrapolate beyond current dynamics and provide robust forecasts of ecosystem responses to future climate change.

This article is part of the theme issue ‘Ecological novelty and planetary stewardship: biodiversity dynamics in a transforming biosphere’.

## 1. Introduction

Ecosystem change results from species response to abiotic drivers and dynamic species interactions. Ecological processes such as dispersal, growth and reproduction occur on a range of timescales, ultimately resulting in complex ecosystem dynamics [1]. While process-based ecosystem models are well-developed [2,3], most forecasts of biodiversity responses to global changes are conducted using species distribution modelling which is based on correlations between current species’ ranges and climate [4]. While these models assume that species demography is at equilibrium dictated by their environmental niche, there are increasing observations that species only partially track their predicted suitable habitat due to transient demographic mechanisms [5,6]. Furthermore, global warming can reshape the structure of communities, notably through the emergence of novel biotic interactions and temperature-dependent competition [3,7]. Forecasts of the future distribution of species and ecosystems are limited by processes that modify rates of change and thus the timescale of the dynamics of the system [8]. Yet, correlation-based, static models are often preferred over their process-based, dynamic counterparts, because of the difficulty of assimilating this data in process-based models, due to limited knowledge of biological processes and rates, and a lack of comprehensive temporal data on whole ecosystems, including multi-species assemblages or communities across trophic levels. We argue that a leap forward in our ability to model the dynamics of whole ecosystems, including the testing of apparent species interactions, is offered by sedimentary ancient DNA (*sed*aDNA).

Palaeo-records such as macro- and microfossils have allowed the documentation of past changes in ecosystems [9–11]. For example, dynamic population models have been fitted to pollen data of four tree taxa to identify the relative importance of temperature change, nitrogen availability and species interaction in determining population dynamics [12]. Similarly, process-based community models with pollen data have been used to study how competition among tree species, density-dependent survival and dispersal rate affect tree abundance [13]. These studies have mainly focused on a handful of species or on shorter time frames, mainly due to the limited taxonomic resolution of pollen data and the scarcity of macrofossils of most species.

*Sed*aDNA records have the potential to overcome the limitations of traditional micro- and macrofossils data [14]. In particular, *sed*aDNA data provide better taxonomic resolution and can recover the abundance of a broader range of species. These include, for example, insect-pollinated forbs, which are generally underestimated in pollen records [15,16], and higher trophic levels like mammals [17–19]. Since *sed*aDNA data contains richer information on past dynamics through time, it has great potential for an improved understanding of the drivers and processes of change. Combining it with process-based models may allow us to improve our forecasts of the effects of ongoing climate change on future biodiversity.

Here, we assess the opportunities of combining *sed*aDNA data with process-based models to study complex ecosystem dynamics under past and future climate changes. We discuss the main advantages of *sed*aDNA data over previous techniques for measuring community change. Aiming at studying biodiversity as a whole, we focus on multicellular organisms, lake sediments and entire communities. Extending beyond plants, our framework can be used to model complete food webs through animal *sed*aDNA. We discuss how complementary data sources can be used, such as climate reconstructions, as well as nitrogen deposition and other global change factors. Finally, we provide a roadmap for the combination of this data with process-based models in order to better quantify the relative importance and rate of biological changes.

## 2. The advantage of *sed*aDNA data

### (a) The nature of *sed*aDNA data

Ancient DNA can be found in a range of substrates like ice cores, permafrost, soils, lake sediments, archaeological artefacts and caves [20]. For time series, it is essential to have a good age–depth model. In general, the most continuous and reliable age–depth models are obtained from lake sediments [21], and therefore we mainly focus on lake sediments here.

Lake *sed*aDNA can be detected in the absence of plant macroscopic remains and is generally thought to derive from DNA binding to clay or other fine particles transported to the lake [14,22]. Whilst some lakes produce poor results due to their sediment composition (acidic bedrocks or algal-dominated gyttja) there is no evidence that changes in natural sediment type has differential effects on the diversity of DNA from plant or animals species, although additions to the sediment, such as bone, may enhance certain taxa [23–25]. It is possible to obtain DNA from ancient pollen using dedicated protocols. However, pollen is in general not the source of *sed*aDNA due to relative low biomass contribution and low chloroplast DNA content [14,26], thus the uncertainties in distinguishing long-distance from local source is avoided. Similar to macrofossil, the *sed*aDNA source area is almost exclusively from within the hydrological catchment and particularly close to the lake, which gives a spatially defined source region [25,26]. This is important for modelling as it means that the *sed*aDNA from even closely located lakes is truly independent, which is not the case with pollen.

**Figure 1.**
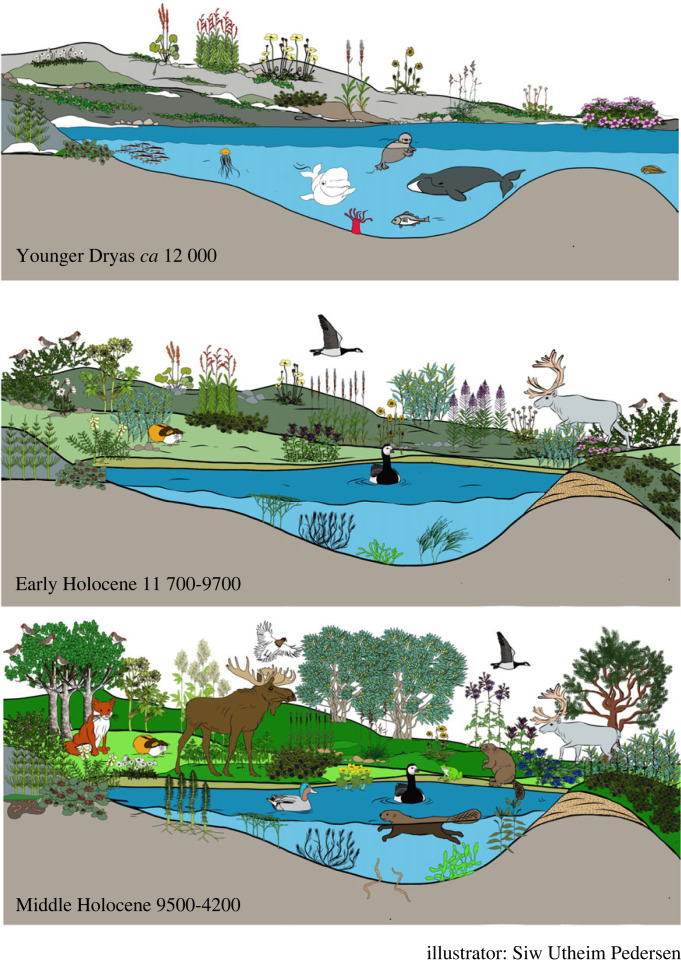
A reconstruction of plants and animals at Nordvivatnet, north Norway, through the last 12 000 years shows examples of species that can be detected in *sed*aDNA studies (drawn after Brown *et al.* [24]). Illustration by Siw Utheim Pedersen.

By sampling lake sediment cores, one can extract DNA and then: (i) amplify the DNA of interest using a primer (a short sequence DNA sequence that serves as a starting point for PCR synthesis) dedicated to the organism group of interest for example mammals or vascular plants (metabarcoding), (ii) ‘capture’ the DNA fragment of interest using capture-probes before sequencing, or (iii) go directly to the sequencing step (shotgun sequencing) which would yield DNA of all organisms in the samples. While the shotgun sequencing has the advantage that it works well even for very old and fragmented DNA, avoids potential bias caused by PCR (see below), and gives simultaneous data on plants and animals, the total gain of target DNA fragments is low and the taxonomic resolution obtainable from metabarcoding studies is not (yet) reached. Because of the higher costs, the time resolution of the few studies undertaken to date is low [17,18,27]. Similarly, only two studies have used broad capture-probes so far, and the taxonomic resolution is low for plants [28,29]. The most commonly used method is metabarcoding, which gives the highest taxonomic resolution for plants [30], and with recent method improvement, also detects mammals well [19,24]. Early studies showed low overlap and poor richness in *sed*aDNA compared to pollen and macrofossil [31,32], but as DNA reference libraries have expanded and molecular methods have improved, the overall richness in *sed*aDNA studies is typically 2–4 times higher than pollen [16,33,34]. Thus, *sed*aDNA may give more complete information about past vegetation than has been possible before, and may also add information about animals in the absence of macrofossils (figure 1).

To identify the DNA fragments obtained, a series of bioinformatic steps are involved, of which the most crucial is matching sequences to a DNA reference library [35]. Both false positives and false negatives occur in *sed*aDNA data, and stringent processes from field to laboratory and bioinformatics are therefore needed [26,36,37]. Conservative data filtering may minimize false positives, but this will always be at the cost of losing true positives [25], so one needs to find a balance. Common reasons for false identification are that closely related taxa may have identical sequences for the marker used, and/or errors in the reference library. The availability of highly curated local DNA reference libraries, such as PhyloAlps or PhyloNorway, reduces the chance of false identification, and may give 40–50% identification to species level [15,19]. As the availability of highly curated reference libraries increases [38], older datasets may be re-analysed to improve identification.

Metabarcoding data provides presence/absence data or quantitative data, and both measures may be biased by the transport and deposition of DNA, the quality of DNA obtained, and technical issues during DNA amplification and bioinformatic analyses [22,25]. For example, the PCR procedure may cause amplification bias due to sequence length and composition, as well as possible mismatch in primer binding sites [25,39]. Repeating the PCR analyses may provide a more robust detection of species [40], and a conservative quantification using these repeats may be advantageous [15,41]. Nevertheless, some taxa such as willows and aquatic macrophytes are commonly assumed to be overrepresented in both metabarcoding and shotgun studies [15,17]. This is likely because their habitat is in/along streams and lakes, and therefore more DNA enters the lake [15,17,25]. However, estimates of ecosystem changes can be quite robust to the quantitative measurement of PCR repeats or proportion of reads, probably because this bias is not changing over time [15].

**Figure 2.**
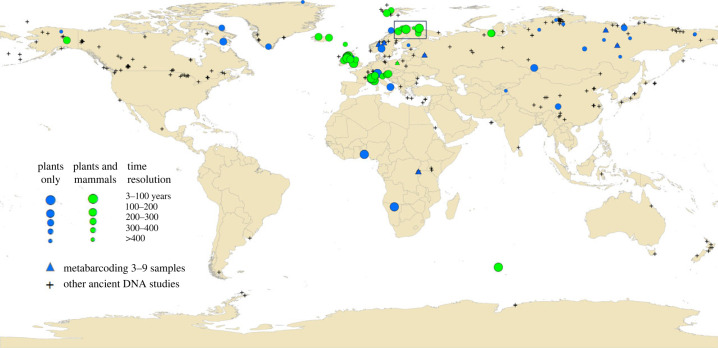
Map of lakes where *sed*aDNA metabarcoding has been analysed for mammals and/or vascular plant. The data is based on von Eggers *et al.* [43] but limited to original metabarcoding studies of lake sediments and updated with relevant papers from the *sed*aDNA society track list (https://sedadna.github.io/ October 2023) and unpublished study sites from our own research groups (see electronic supplementary material, table S1). Square denotes lakes used in figure 3. To show the broader potential to obtain ancient DNA data from other regions, all sites from [43] are shown as crosses except when sediment type was cave, lake surface, marine surface, midden, sediment trap or soil, and except sites that lacked age or had age = 0 (except for permafrost sediments and sites in Wang *et al.* [17] which we assume are ancient).

Metabarcoding is now approaching standardization across different laboratories, which allows dataset pooling [41,42]. There are high-resolution metabarcoding data available from mainly arctic and alpine sites, where also most ancient DNA studies in general have been done (figure 2). However, studies from some warmer regions show promising results: for example, in Italy [44] where Holocene sediments generally provide good DNA quality and some DNA was obtained from up to 31 000 years ago; an African savanna site with good DNA over the last 170 years [45]; and an early study on an African high altitude site with up to 5000 year old DNA [46]. There is a large metabarcoding circum-Arctic study of 21 sites and 242 samples [47], but this is based on permafrost. The largest lake sediment metabarcoding study is one of 10 northern Fennoscandian lakes (387/355 samples x 8 PCR replicates, [15,41]), and a study of 705 samples from 14 lakes in the European Alps will soon be available (figure 2; electronic supplementary material, table S1). There is also an increasing number of lakes analysed from Siberia and the Tibetan Plateau [34,48] that generally cover long time spans (up to about 50 kyr), and many shorter records from the British Isles [49,50] (figure 2, electronic supplementary material, table S1). By using quality measures and standardization [41], the dataset may be combined into even larger datasets.

In association with *sed*aDNA data, complementary abiotic and biotic information can be obtained from the sediment core. It is common to make other simultaneous measurements such as X-ray fluorescence (XRF), magnetic susceptibility, stable isotopes (N and C) and biomarkers [51–53]. In addition, independent climate reconstructions such as CHELSA [54], NGRIP [55], oxygen isotope [56,57] or chironomids may be used [19], although, as with all palaeoproxies, one has to account for uncertainties due to different spatial and temporal resolution [58]. These provide important information about changes in the environment. Thus, by combining *sed*aDNA data with other proxies, we are moving towards the reconstruction of both abiotic changes and species composition which allows studies of interactions, food webs and, ultimately, whole-ecosystem changes over time.

The dynamics of ecosystems are influenced by natural and anthropogenic environmental changes, which can include a variety of abiotic factors such as temperature, precipitation and soil characteristics. Biotic interactions regulate the coexistence between species and play a critical role in shaping ecosystem changes [3,59]. The interactions between species can take many forms, including competition for resources such as food, water and shelter, predation, facilitation, inhibition, mutualism and parasitism [60,61]. Understanding how these interactions affect the changes in species abundance and distribution, and how they are themselves influenced by abiotic factors, is essential for predicting the response of ecosystems to all environmental changes [3].

Direct quantification of biotic interactions can be a challenging task, but inverse modelling techniques can be used to extract the signatures left by biotic interactions on past ecosystem dynamics. *Sed*aDNA time series are highly relevant in this task. The majority of metabarcoding studies have focused on plants and microbes, but an increasing number of studies also include other organisms such as mammals [22,43]. Early metabarcoding methods gave scattered records of mammals [47] or provided good detection only of domestic mammals, which are commonly at high densities [49,62]. It has generally been challenging to obtain mammal DNA from sediments [63] for two reasons: (i) the abundance of mammal DNA is lower and more patchy than for plants, and (ii) co-amplification of human DNA during PCR [63,64]. Co-amplification of human DNA was partly overcome by adding small fragments of DNA that bind to human DNA, so-called blocking oligos, on both forward [62] and, more recently, reverse primers [19]. Using both blocking oligos increases detection of mammals and also co-amplifies birds, fish, amphibians and worms (figure 1). Additional organisms, such as diatoms, fungi and lichens, can be amplified through the use of dedicated primers [65–67], giving a broader spectrum of biodiversity. Having multiple trophic levels allows a more comprehensive tracing of ecosystem dynamics.

The high taxonomic resolution obtained in recent *sed*aDNA studies of plants allows the assigning of traits that provide a window on ecosystem characteristics and the function of species. Species-level information allows linking of pollinator dependence, nutrient demands, ability to compete with established vegetation, and dispersal mode [15]. For example, certain plants may inform us about past human land-use and its effects on biodiversity [19,50,62]. Further, soil disturbance, temperature optimum and moisture values can be estimated from plant traits, and be shown to be correlated with local glacial activity [68]. The high taxonomic resolution obtained from northern Fennoscandia allowed the combining of 227 of 238 vascular plant taxa with the Swedish plant trait database of 30 traits [15,69]. Also, species-level information on mammals can be used to reconstruct trophic interactions linking species through prey–predator dynamics or food webs. Thus, by linking species to traits, one can reconstruct past external drivers such as human or glacial activity. Furthermore, our understanding of how traits relate to one another can be increased and we can gain functional understanding of internal ecosystem dynamics. Traits can also be used to reduce the dimensions in ecological equations and thereby reduce ecosystem model complexity [70].

### (b) Temporal resolution and processes

The processes that shape ecosystem dynamics occur over much longer time scales than the few decades over which we have been monitoring ecosystems. In the most extreme cases, legacies of the last glaciation are still found in current species distribution patterns [71–73]. As regards modern time series, most span only a few decades and even the best resolved time series are usually yearly and focus on only one taxonomic group [74]. Additionally, the temperature changes that are predicted to occur over the next few decades have only occurred over millennial timescales in the past. Therefore, it is crucial to use long-term ecosystem time series for the calibration of models. It is important to recognise that all ecosystem processes occur at differential rates, and some may occur quite rapidly. For example, competitive exclusion can have a significant effect on an ecosystem and may happen over just a few decades. Therefore, high-resolution time series are essential for accurately capturing the dynamics of these processes. The use of *sed*aDNA data is a promising approach for obtaining longer-term ecosystem time series. However, it remains to be seen how far we can extend these records and how well they can capture the full range of ecosystem processes. High temporal resolution (on average every 1.3–4 years) is available from short-term studies covering a few decades up to three centuries [26,45,67]. For millennial-scale studies, the highest temporal resolutions we are aware of are on average every 53 [23] and 64 years [15,41], but there are large numbers of sites published or in preparation with time resolution of decades to a few hundred years (figure 2; electronic supplementary material, table S1). Dense sampling across rapid climatic changes such as the Bølling/Allerød-Younger Dryas-Holocene boundaries at every 87 years, are particularly relevant for ongoing studies of rapid climate changes [75].

**Figure 3.**
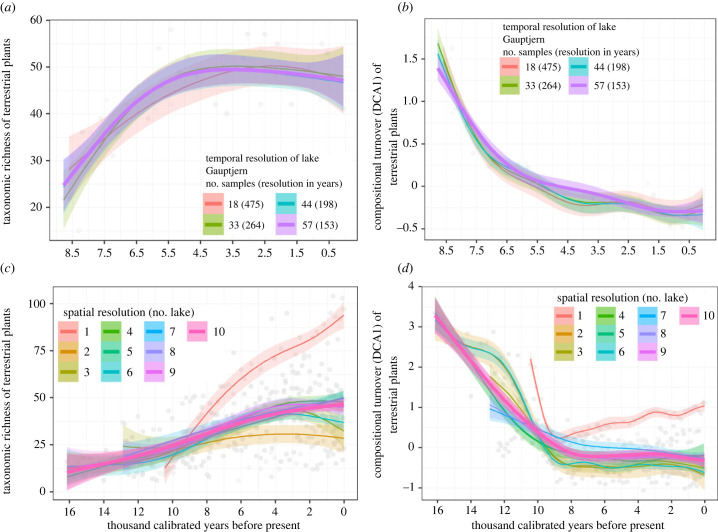
Impact of temporal and spatial resolution on richness and compositional turnover detected. Effect of temporal sample resolution at one lake (lake Gauptjern, 8.5–0 cal BP, figure 2) on (*a*) taxonomic richness and (*b*) compositional turnover. Effect of spatial resolution using 1 to 10 lakes on (*c*) taxonomic richness and (*d*) compositional turnover. Data from Alsos *et al.* [15]. Coloured shadings indicate 95% confidence interval of the fitted values of the generalised additive models (GAM).

To explore the minimum density of samples needed to obtain a representative temporal resolution, we sub-setted one to three samples from each 500 years bin from Alsos *et al.* [15]. We used lake Gauptjern which had mean temporal resolution of 153 years over the last 8.5 kyr. When 18, 33 and 44 samples (resulting in mean temporal resolution 475, 264 and 198 years, respectively) were re-analysed, both the taxonomic richness and compositional turnover of all sampling frequencies showed similar and comparable patterns to the full core (figure 3*a,b*). However, although not significant, we do note that both parameters may be underestimated for some periods, e.g. around 5.5 and 4.5 ka. We also note that this is a boreal region with less fluctuating pattern than for example seen in the Alps [19]. Thus, we advocate a minimum temporal resolution of every 150–260 years.

### (c) Toward a denser network of sediment records for spatial processes

The temporal dynamics of species within an ecosystem may switch between internal processes and external factors, such as dispersal from other ecosystems [76]. Local ecological systems are connected to form meta-communities that exchange species. The addition of new species through dispersal can bring new ecological traits that can alter the abundance of species and the overall ecological trajectory of the ecosystem [15]. For example (i) the arrival of tree species generally causes a major change to arctic vegetation [77], (ii) the reintroduction of wolves has had a significant impact on the ecological dynamics of Yellowstone [78], and (iii) beaver, which is well recorded in *sed*aDNA [24], has profound effects on temperate and boreal ecosystems [79]. This highlights the importance of studying both the internal dynamics of an ecosystem and its interactions with other ecosystems in order to fully understand its functioning. In ecological research, there has traditionally been a trade-off between spatial and temporal resolution, with studies often focusing on one or the other. However, increased availability of high taxonomic and temporal resolution *sed*aDNA data makes it possible to simultaneously study both spatial and temporal dynamics of ecosystems through time. By analysing sediment samples from lakes, researchers can obtain millennia-long time series of species composition, providing a unique window into the spatial dynamics of these ecosystems. While some lakes have been studied using this technique, the vast majority remain uninvestigated and represent a wealth of untapped information about the history of ecosystems. Expanding the use of *sed*aDNA analysis to more sites, especially to regions currently underrepresented (figure 2), will generate a better understanding of the ecosystem change at a range of temporal and spatial scales.

To determine past dispersal rates, spatially and temporally high resolution data are required. The spatial resolution of *sed*aDNA studies is far lower than currently available for pollen, with only about 100 lakes analysed for plant *sed*aDNA [42,43] (figure 2). However, even a scattered network of sites allows the production of past distribution maps and the calculation of minimum dispersal rates, as has been done for tree species based on pollen [80,81]. As there is currently no or only scattered fossil information on the majority of species, this already has provided a major increase in knowledge that can inform models.

The number of *sed*aDNA studies is increasing rapidly [22,82], and for some regions, average distance among sites is low, allowing the estimation of regional post-glacial arrival patterns of species as well as relative importance of abiotic and biotic factors in determining arrival times [15]. Furthermore, such data can be used to calculate the time from arrival to local spread, allowing the possibility of incorporating a colonisation term into the models. The dataset also allows us to test how many study sites within a region are needed to detect consistent spatial patterns. Based on our estimates of the effect of spatial resolution on species richness and turnover (figure 3*c,d*), *sed*aDNA is starting to provide consistent temporal patterns if a broader time period (e.g. 500-year bin) is represented in two lakes. For example, temporal trends of both taxonomic richness and compositional turnover remain comparable (figure 3*c,d*) at the regional scale in northern Fennoscandia, when a 500-year bin is represented by one random sample each from two randomly chosen lakes, indicating that as few as two lakes can be sufficient to capture regional trends of diversity. Thus, for some regions, the available data already provides sufficient coverage to infer both spatial and temporal variation using a meta-community framework, while for more heterogenous regions, more sites may be required.

## 3. Confronting dynamic models with *sed*aDNA data

### (a) Modelling ecosystem trajectories

Development and validation of models are necessary to obtain a more comprehensive understanding of the ecosystem changes, and a good model will eventually permit robust predictions [83]. Two modelling paradigms are commonly used to model biodiversity. On the one hand, correlative models, such as species distribution models (SDMs, [4,84]), are derived from statistical patterns extracted from the data and deriving relationships between species presence (or abundance) and environmental covariates (e.g. temperature and precipitation). Predictions with correlative models assume that potentially very complex [85] patterns contained in the data will repeat [86]. On the other hand, process-based models explicitly account for the processes underlying ecosystem dynamics [87]. Process-based models rely on data for their calibration and the estimation of the initial state of the system but are built together with *a priori* scientific knowledge about the system [88]. The process knowledge embedded in their structure renders them more robust for predicting the future trajectories of ecosystems under novel ecological conditions [87]. Process-based models are theoretically superior to correlative models since statistical patterns observed at time *t* may not hold at time *t* + 1 because of ecological feedbacks, which trigger large shifts in ecosystem states [59,86]. Moreover, process-based models can account for both abiotic factors and interactions between species, which can provide a more accurate representation of how biodiversity will respond to changes in their environment [86].

Correlative models are nonetheless the most used approach due to their sound statistical basis and practicality [89]. *Sed*aDNA data may be used in combination with palaeoclimate models to validate species presence back in time, as has also been done with macrofossil and pollen [90,91]. Modelling efforts with *sed*aDNA data have included individual-based models and simulations under climate warming [92,93]. To our knowledge, *sed*aDNA has yet to be used for the purpose of differential equation-based modelling, which has the advantage of being much more scalable than other process-based modelling approaches [94].

**Figure 4.**
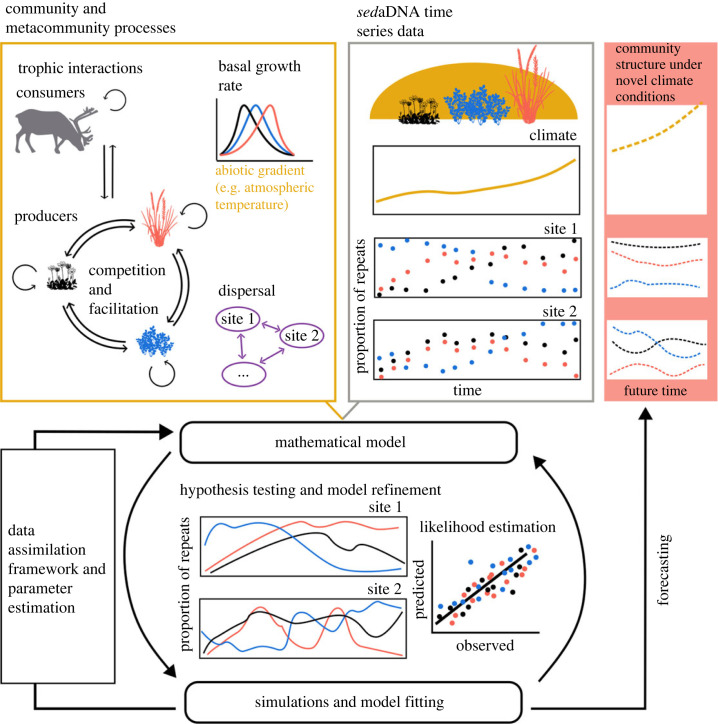
Fitting a general dynamical community model of three plant species using *sed*aDNA data. Here, the dynamics of species (blue, red, black) at two sites depend on the species' basal growth rates, which are differentially affected by abiotic environmental factors such as temperature, as well as interactions between species and dispersal of species between sites. Climate models can provide estimates of past temperature while the *sed*aDNA time series data itself (here illustrated as change in abundance [proportion of repeats]), supported by external input (expert knowledge, experimental data, etc.), can be used to fit the parameters of complex and competing models in order to best describe the past dynamics of biotic communities as well as project future community dynamics under novel environmental conditions.

A process-based dynamical model describes how the population of a particular species changes over time in an ecosystem (figure 4). Denoting the abundance of a species *i* at site *k* by $N_{i}^{k}$, a dynamical community model generally takes the form:

3.1 $$\overset{p.c.\;\mathrm{growth}\;\mathrm{rate}}{\overbrace{\frac{1}{N_{i}^{k}}\frac{d}{dt}N_{i}^{k}}}=\underset{\mathrm{basal}\;\mathrm{growth}\;\mathrm{rate}}{\underbrace{b_{i}(u^{k}(t))}}-\sum_{\;j=1}^{S}\left[\overset{\mathrm{competition}\;\mathrm{or}\;\mathrm{facilitation}}{\overbrace{\alpha_{i,j}N_{j}^{k}}}+\underset{\mathrm{trophic}\;\mathrm{interactions}}{\underbrace{\varepsilon {\left[F(N)\right]}_{i,j}N_{j}^{k}-[F{\left(N^{k}\right)}_{i,j}N_{j}^{k}]}}\right]+\underset{\mathrm{dispersal}}{\underbrace{\sum_{l=1}^{L}m_{i}^{k,l}N_{i}^{l}-N_{i}^{k}\sum_{l=1}^{L}m_{i}^{l,k}}},$$

where $N^{K}=(N_{i}^{k},\ldots ,N_{S}^{k})$ is a vector containing species abundance at site *k*. Equation (3.1) relates the per capita growth rate of species, $1/N_{i}^{k}\;dN_{i}^{k}/dt$, to the biotic and abiotic processes that affect them. These processes generally comprise reproduction, captured by the basal growth rate *b_i_* which may depend on time-dependent environmental drivers $u^{k}(t)$. They further include biotic interactions that depend on the abundance of other species $N_{j}^{k}$, which comprise competition and facilitation, captured by the coefficient *α_i,j_* measuring the competition or facilitation strength between species *i* and species *j*, and comprises trophic interactions, captured by the functional response *F*. Lastly, they can include spatial processes, i.e. dispersal across locations, where $m_{i}^{k,l}$ is the dispersal rate for species *i* from site *k* to site *l*. By explicitly accounting for biological processes, equation (3.1) constrains the model predictions. For instance, equation (3.1) predicts that the growth rate of a species decreases with increasing competition from other species, as captured by the competition coefficient *α_i,j_*. Also, with spatial dispersal ($m_{i}^{k,l}>0$), the model allows some metapopulation dynamics, as the abundance of a species at one location can influence its abundance at other locations. Dynamical models are simulated over time by numerically integrating equation (3.1). This requires an initial condition estimate of the system, which together with the model parameter values will determine the ecological trajectory.

### (b) Inverse modelling

The parameters and processes within process-based models have traditionally been determined independently of the model, with empirical data only used for model validation and comparison [95]. Independent estimation of parameters and processes rapidly becomes impossible as the number of species modelled increases, especially when considering species interactions, dispersal, etc. Inverse modelling, which consists in using observation data to recover the parameters of a model that can best explain the data, allows the bridging of this gap [95,96]. In particular, the *sed*aDNA observations contain signatures of underlying biological processes that have shaped the dynamics of ecosystems, and can therefore be used to parameterize the model. These signatures may be tapped by inverse modelling methods to recover the processes by matching model predictions and data [97]. Different approaches may be used for this process, such as Bayesian methods or variational methods, all of which rely upon the estimation of the likelihood of the model parameters given the data [98,99]. The complexity of the models, which mirrors the intricacies of biotic processes, should be thoughtfully selected in alignment with the quality and quantity of *sed*aDNA data—a principle reminiscent of Ockham's razor [83]. In reducing model complexity, trait-based transfer functions emerge as a valuable tool, streamlining the modelling process [70].

The interpretation of the calibrated model parameters can provide information on the ecological processes taking place and advance ecological theory. For instance, the sign and absolute value of the competition/facilitation interaction term (*α*) in equation (3.1) informs about the interactions between species. To effectively interpret inferred parameters, it is essential to account for uncertainty estimates. Bayesian methods excel in explicitly handling uncertainties when compared to variational methods, but they do come with a notable computational overhead. A prudent approach involves generating synthetic data by simulating information using a model with known parameters [100,101]. This step serves as a crucial validation process to confirm the model's ability to successfully extract and reflect biotic processes present in the real data. Moreover, prior information on biotic processes, whether derived from expert opinion [102,103], experimental data or other data sources [2] can be useful to help characterise biotic processes with weak or complex signatures. For communities with no extant analogs, the repetition of inferred biotic interactions across independent sites can provide greater support for causal inference. Inverse modelling may be further used for hypothesis testing. In such cases, alternative models corresponding to the different hypotheses are formulated, and inverse modelling is used to evaluate the likelihood of each model [104].

The calibrated model may eventually be used to make predictions about future ecosystem dynamics under different environmental change scenarios. Future trajectories of the ecosystem may be simulated, for instance to predict how the abundance of different species might change under climate change [105]. By comparing the predicted dynamics under different scenarios, ecologists can gain insights into how ecosystems might respond to different environmental stressors and inform conservation and management strategies.

## 4. Future perspectives

The combination of *sed*aDNA data with process-based models offers a unique approach to understanding past ecosystem dynamics and how they may respond to global changes. The reliable detection of taxa still represents a challenge for *sed*aDNA studies. To obtain better data, especially for rare species, one could add more technical replicates as well as increasing sampling density both in time and space, and so reducing the rate of false negatives [25]. This would better inform process-based models and make the use of *sed*aDNA even more relevant to species of conservation concern. Also, the current paper focuses mainly on plants and mammals from metabarcoding data, as these are the most readily available now and best studied in terms of *sed*aDNA, present ecology and modeling. For other organism groups, it would be valuable to compare *sed*aDNA data with an independent record of species occurrence, to know how well the species are represented in the *sed*aDNA data [25]. Further improvement is offered by (i) improved DNA reference libraries to ensure correct identification, (ii) multiplexing and other method development to detect more organism groups from the same samples, and (iii) more sites investigated, especially for ecosystems currently underrepresented in *sed*aDNA studies. *Sed*aDNA data are generally available in raw format in data repositories such as Dryad. An important step will be to make the *sed*aDNA data available in an easily accessible format such as the final species list, for example in Neotoma [42], which can be combined with other databases of for example traits. Thus, we expect that in the near future, *sed*aDNA will be commonly used in combination with process-based models to forecast future biodiversity. With *sed*aDNA that contains rich information on past dynamics, modellers can at last test and refine their biodiversity models and theory at the species, community or ecosystem level. This is a major step forward, given that ecological modellers have until now barely confronted model predictions against data, and were largely confined to theoretical predictions.

An important postscript is that our approach outlined above relies on the assumption that traits observed in the current time were also operating in the past. This might not be the case if species currently are restrained by interactions that did not take place in the past. However, using independent proxies, niche stability through time may be explored. As more metagenomic data becomes available, one can imagine obtaining functional genes directly [106] and thereby omitting the step of species and traits. However, this would also require knowledge on how genes relate to the environment, which is an exciting emerging avenue.

## Data Availability

Data used in figure 3 are available in Alsos *et al*. [15]. The data used in figure 2 are provided in the electronic supplementary material [107].
